# Clinical efficacy and safety of venetoclax combined with hypomethylating agents in relapsed high-risk acute myeloid leukemia patients after allogeneic hematopoietic stem cell transplantation

**DOI:** 10.3389/fmed.2025.1638176

**Published:** 2025-09-15

**Authors:** Jiaying Cheng, Haipeng Fu, Ling Jiang, Yun Huang, Yujiao Zhang, Zhiquan Long, Xuejie Jiang

**Affiliations:** Department of Hematology, Nanfang Hospital, Southern Medical University, Guangzhou, China

**Keywords:** myeloid neoplasia, cytotoxic regimens, therapeutic resistance, disease recurrence, venetoclax

## Abstract

**Introduction:**

Relapse after allogeneic hematopoietic stem cell transplantation (allo-HSCT) for high-risk myeloid malignancies remains a major therapeutic challenge, with conventional chemotherapy offering limited survival benefits. BCL-2 inhibition combined with hypomethylating agents (HMAs) has emerged as a potential therapeutic option, but comparative data in this setting are scarce.

**Methods:**

We conducted a single-center retrospective study of 106 consecutive patients with post-transplant acute myeloid leukemia (AML) recurrence treated between 2020 and 2024. Patients received either venetoclax plus HMAs (*n* = 53) or intensive chemotherapy (*n* = 53). Outcomes assessed included complete remission (CR) rate, overall survival (OS), measurable residual disease (MRD) clearance, and treatment-related toxicities. Multivariable Cox regression analysis was performed to evaluate survival predictors.

**Results:**

The venetoclax-based regimen achieved significantly higher CR rates (56.6% vs. 26.4%, *p* = 0.002) compared with intensive chemotherapy. Median OS was markedly improved with venetoclax plus HMAs (12.6 vs. 5.8 months; HR 0.42, *p* < 0.001). MRD clearance was more frequent in the venetoclax group (70.0% vs. 35.7%, *p* = 0.021). Safety analysis demonstrated lower incidences of severe cytopenias (36.8% vs. 64.2%, *p* = 0.002) and infectious complications (11.3% vs. 32.1%, *p* = 0.008). Multivariable modeling confirmed venetoclax-based therapy as an independent predictor of improved survival (adjusted HR 0.42, 95% CI 0.31–0.58).

**Discussion:**

Venetoclax in combination with HMAs provided superior clinical benefits over intensive chemotherapy in post-allo-HSCT AML relapse, achieving higher remission rates, improved survival, enhanced MRD clearance, and a favorable safety profile. These findings highlight venetoclax-based regimens as a promising therapeutic approach for this high-risk population.

## 1 Introduction

Acute myeloid leukemia (AML) represents a molecularly heterogeneous and clinically aggressive hematologic malignancy characterized by rapid clonal proliferation of myeloid precursors. This has consistently remained a focal point in hemato-oncology research due to the persistent need for optimized therapeutic strategies (1). Although allogeneic hematopoietic stem cell transplantation (allo-HSCT) cures 40–50% of high-risk AML patients (2), relapse remains the leading cause of death. As a result, the 3-year survival rate after early recurrence is less than 10% (3). A 2023 meta-analysis of 1,852 post-HSCT relapses revealed 12-month overall survival (OS) rates of 15–28% with conventional therapies, highlighting a need for better treatment options (4). This pressing clinical reality underscores the limitations of current therapeutic approaches and drives the ongoing pursuit of more effective salvage regimens.

Among existing treatment options, conventional chemotherapy protocols, such as fludarabine plus cytarabine (FLAG) or cladribine combined with cytarabine (CLAG), can induce remission in some patients; however, these treatments demonstrate modest complete response rates of only 25–35%, alongside treatment-related mortality rates reaching 20–30% (5, 6). Notably, their efficacy is even more constrained in patients harboring adverse genetic profiles, including complex karyotypes or TP53 mutations. Emerging immunotherapies—encompassing donor lymphocyte infusion (DLI) and bispecific antibodies—show promising potential, although their clinical utility remains hampered by graft-versus-host disease (GVHD) risks and accessibility barriers (7, 8). While second transplants may offer durable remission for a subset of patients, procedural toxicities and donor availability remarkably restrict their applicability.

**Figure 1:**
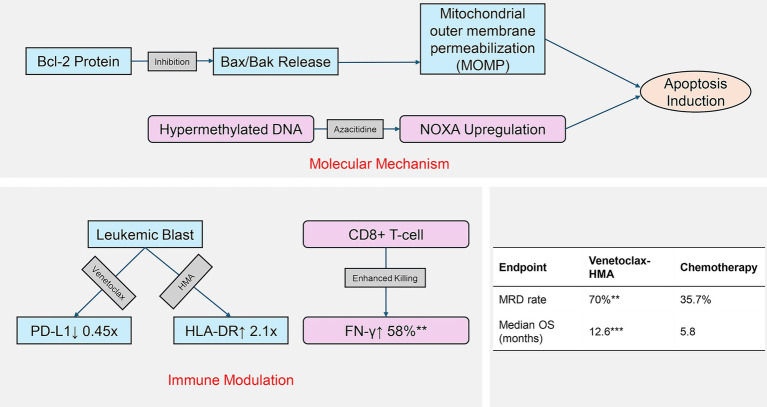
Dual mechanism of venetoclax-HMA in post-transplant relapse. Molecular pathways: venetoclax inhibits BCL-2 to activate apoptosis, while HMA reverses epigenetic silencing. Immune modulation: combined PD-L1 downregulation and antigen presentation enhance T-cell recognition. Clinical outcomes reflect mechanistic synergy.

The evolving understanding of AML pathogenesis has catalyzed the development of targeted therapies, with the BCL-2 inhibitor venetoclax marking a therapeutic milestone (9, 10). By selectively binding to the BCL-2 protein, venetoclax restores the apoptotic capacity in leukemic cells. When combined with hypomethylating agents (HMAs), it has demonstrated groundbreaking efficacy in elderly AML patients who are not suitable for intensive chemotherapy, achieving a complete remission rate of 60–75% (11). Mechanistic studies have further revealed that venetoclax may potentiate T-cell anti-leukemic activity via PD-1/PD-L1 pathway downregulation, while HMAs enhance tumor antigen presentation through epigenetic modulation (12). This dual mechanism holds particular promise in post-transplant relapse cases characterized by a unique immune microenvironment. Preclinical evidence has also highlighted the selective targeting of leukemia stem cells by this regimen, potentially underpinning its sustained therapeutic benefits. As illustrated in Figure 1, the regimen’s efficacy stems from venetoclax-mediated restoration of mitochondrial apoptosis, coupled with HMA-driven epigenetic reprogramming of leukemic stem cells.

However, critical knowledge gaps persist regarding venetoclax-HMA combination therapy for relapsed AML following allo-HSCT. Published studies have predominantly included limited cohorts (typically <50 patients) and have often lacked comprehensive long-term follow-up (13, 14). Notably, post-transplant immune reconstitution and marrow function differ markedly from those with *de novo* AML. These factors may substantially influence drug metabolism and treatment response. In addition, therapeutic outcomes vary significantly across molecular subtypes: TP53 mutations confer chemoresistance via apoptotic pathway disruption, FLT3-ITD drives survival through STAT5 hyperactivation, and RAS mutations promote proliferation via MAPK signaling. In addition to genetic alterations, non-genetic adaptations—including changes in the ratios of BCL-2/MCL-1 proteins, OXPHOS metabolic dependency, and differentiation blockade—further contribute to therapeutic resistance (4, 5). These complexities underscore the need for multimodal targeting strategies. Addressing these questions is paramount for refining clinical decision-making.

We conducted a large-scale (*n* = 106), single-center retrospective cohort study to holistically clarify the clinical merits of venetoclax-HMA in post-allo-HSCT relapsed AML. Beyond conventional efficacy endpoints, we placed particular emphasis on correlating molecular markers with treatment response and assessing inter-subgroup benefit disparities. Concurrently, this research meticulously examined the safety profiles of this specialized population, including the impacts on hematopoietic recovery and GVHD risks. The accrued dataset offers vital insights for individualized therapeutic planning and establishes a foundation for subsequent prospective research.

## 2 Materials and methods

This research used a single-center retrospective cohort design and was conducted in strict compliance with the ethical principles outlined in the *Declaration of Helsinki*. The study protocol was approved by the Ethics Committee of Southern Medical University (Approval #NFEC-2024-271, September 2024). Written informed consent was obtained from all participants enrolled in the study.

### 2.1 Patient cohort

Medical records from the hematology department of our hospital were systematically reviewed between January 2020 and December 2024. The inclusion criteria were as follows: (1) age ≥18 years; (2) morphologically, immunophenotypically, and molecularly confirmed AML (WHO 2022 criteria); (3) first documented bone marrow or extramedullary relapse following allo-HSCT; (4) ECOG performance status ≤2 points; and (5) availability of complete clinical follow-up documentation. The exclusion criteria were as follows: (1) active GVHD requiring intensive immunosuppression; (2) prior exposure to venetoclax or HMA agents; (3) significant cardiac, hepatic, or renal dysfunction (LVEF <50%, Child–Pugh class B/C, or CrCl <30 mL/min); (4) pregnancy or lactation; and (5) history of other malignancies. Ultimately, 106 eligible participants were enrolled and assigned to two groups through propensity score matching (PSM) in a 1:1 ratio, resulting in venetoclax + HMA (*n* = 53) and chemotherapy (*n* = 53) cohorts. Matching variables included age, sex, ELN2017 risk stratification, and pre-transplant disease status, and comprehensive clinical and molecular data were available for all study endpoints.

### 2.2 Treatment measures

The venetoclax + HMA regimen consisted of oral venetoclax (AbbVie) at a dosage of 400 mg daily (600 mg for BSA ≥1.8 m^2^) over 28-day cycles. This was combined with either subcutaneous azacitidine (Celgene) at a dose of 75 mg/m^2^ (days 1–7) or intravenous decitabine (Chia Tai Tianqing) at a dose of 20 mg/m^2^ (days 1–5). The chemotherapy regimen included the following: FLAG: fludarabine 30 mg/m^2^ + cytarabine 2 g/m^2^ IV (days 1–5) with Granulocyte Colony-Stimulating Factor (G-CSF) 5 μg/kg SC (initiated 24 h pre-chemotherapy and continued until neutrophil recovery); CLAG: cladribine 5 mg/m^2^ + cytarabine 2 g/m^2^ IV (days 1–5) with identical G-CSF administration. Standard infection prophylaxis included levofloxacin (500 mg/day), acyclovir (400 mg bid), and fluconazole (200 mg/day) until the absolute neutrophil count (ANC) exceeded 0.5 × 10^9^/L.

All interventions continued until disease progression, unacceptable toxicity, or completion of a maximum of six cycles. Disease progression was defined as any of the following: (1) >50% increase in bone marrow blasts, (2) new extramedullary lesions, or (3) peripheral blood blasts >5%. Unacceptable toxicity included the following: (1) grade 4 non-hematologic toxicity (Common Terminology Criteria for Adverse Events (CTCAE) version 5.0), (2) grade 3 cardiac/pulmonary toxicity, or (3) treatment delay >21 days due to adverse events.

### 2.3 Efficacy evaluation system

Efficacy was evaluated according to the 2022 European LeukemiaNet (ELN) criteria, with the following primary endpoints: complete remission (CR): bone marrow blasts <5%, no extramedullary disease, ANC ≥1.0 × 10^9^/L, and a platelet count of ≥100 × 10^9^/L; complete remission with incomplete hematologic recovery (CRi): meeting all CR criteria except for incomplete hematologic recovery; and partial remission (PR): bone marrow blasts ≥50%, resulting in a range of 5–25%. Leukemia-associated immunophenotypes (LAIPs) were assessed using qPCR for fusion transcripts (PML-RARA, CBFB-MYH11) and mutations (NPM1, FLT3-ITD). Minimal residual disease (MRD) was analyzed via 8-color flow cytometry (FACS Canto II, BD Biosciences; sensitivity 10^−4^). CR/CRi required confirmation through two consecutive bone marrow assessments that were conducted at least 4 weeks apart. MRD negativity was defined as <0.01% leukemic cells within 30 days of achieving CR.

### 2.4 Survival follow-up and safety monitoring

Survival analyses were conducted following ITT principles, focusing on two primary endpoints: OS, defined as the time from relapse to death or last follow-up, and relapse-free survival (RFS), which measures the duration from CR achievement to relapse, death, or last follow-up. Monthly evaluations included hematologic parameters, bone marrow examinations, and imaging when indicated. Safety was graded according to the CTCAE (version 5.0): hematologic toxicities (neutropenia, anemia, and thrombocytopenia), non-hematologic events (infections, hepatotoxicity, and GI disturbances), and GVHD exacerbation (using the NIH consensus criteria). All adverse events were documented for up to 30 days post-treatment.

### 2.5 Statistical analysis

The data were analyzed using SPSS 27.0 and GraphPad Prism 9.0. PSM was performed using 1:1 nearest-neighbor matching with a caliper width of 0.2 standard deviations of the logit score. The proportional hazard assumption was validated via Schoenfeld residual testing (all *p* > 0.05), and time-dependent covariates were excluded after confirming non-significance. The covariates included age (±5 years), ELN2017 risk stratum, pre-HSCT MRD status, and time to relapse (±30 days). Balance was assessed using standardized mean differences (<0.1 considered adequate). Subgroup analyses were performed using the Benjamini–Hochberg false discovery rate (FDR) correction. Only an adjusted *p*-value of <0.1 was interpreted. Normally distributed continuous variables were expressed as mean ± SD (independent *t*-tests). A post-hoc power analysis using GPower 3.1 indicated 78% power to detect HR = 0.42 at *α* = 0.05 with *n* = 106, reducing the risk of type II error to 22%. Sensitivity analysis confirmed detectable effect sizes ≥0.38 with the current *N*. Categorical variables were expressed as counts (%) (*χ*^2^/Fisher’s exact tests). Survival curves (Kaplan–Meier) were compared using log-rank tests. Multivariate Cox regression analyzed the effects of treatment regimen, age, ELN risk, and pre-HSCT MRD status. All tests were two-tailed, with a significance level defined at *α* = 0.05.

## 3 Results

### 3.1 Comparison of the baseline characteristics of relapsed high-risk AML patients after allo-HSCT

After PSM, the baseline characteristics of the two groups were well balanced (Table 1). The median age in the venetoclax + HMA group was 50 years (range: 21–73), while in the chemotherapy group, it was 52 years (range: 23–71), with no statistical significance (*p* = 0.385). In terms of AML characteristics, the proportion of high-risk ELN2017 patients between the two groups was similar (73.6% vs. 69.8%, *p* = 0.672), and there was no significant difference in the pre-transplant MRD positivity rate (39.6% vs. 35.8%, *p* = 0.694). It is worth noting that the two groups were comparable in terms of transplant type (fully matched/haploid/unrelated), pre-treatment regimen (myeloablative/reduced intensity), and GVHD prevention regimen (*p* > 0.05).

**Table 1:** Baseline characteristics of relapsed high-risk AML patients after allo-HSCT.

Clinical characteristics	Venetoclax-HMA (*n* = 53)	Chemotherapy (*n* = 53)	*p*-value	SMD
Age (years), median [range]	50 [21–73]	52 [23–71]	0.385	0.07
Male patients, *n* (%)	32 (60.4)	29 (54.7)	0.683	0.11
ECOG PS 0–1, *n* (%)	45 (84.9)	43 (81.1)	0.602	0.09
ELN2017 high-risk, *n* (%)	39 (73.6)	37 (69.8)	0.672	0.08
Secondary AML, *n* (%)	13 (24.5)	9 (17.0)	0.341	0.18
Pre-HSCT MRD+, *n* (%)	21 (39.6)	19 (35.8)	0.694	0.07
Donor type			0.843	
Matched related, *n* (%)	28 (52.8)	26 (49.1)		0.07
Haploidentical, *n* (%)	18 (34.0)	20 (37.7)		0.08
Unrelated, *n* (%)	7 (13.2)	7 (13.2)		0.00
Conditioning intensity			0.912	
Myeloablative, *n* (%)	35 (66.0)	34 (64.2)		0.04
Reduced-intensity, *n* (%)	18 (34.0)	19 (35.8)		0.04
Lines of therapy, median [range]	2 [1–4]	2 [1–5]	0.820	0.05
GVHD prophylaxis			0.910	
Tacrolimus/MTX, *n* (%)	42 (79.2)	41 (77.4)		0.04
Cyclosporine/MMF, *n* (%)	11 (20.8)	12 (22.6)		0.04
Early relapse (≤12 months), *n* (%)	31 (58.5)	29 (54.7)	0.701	0.07

Propensity score matching (1:1) was performed with a caliper width of 0.2 SDs of the logit scores. The matching covariates included age (±5 years), sex, ELN2017 risk, pre-HSCT MRD status, time to relapse (±30 days), and prior therapy lines. Statistical tests: continuous variables (the Mann–Whitney *U* test); categorical variables (McNemar’s test for binary; the Stuart–Maxwell test for multicategory). Balance assessment: standardized mean differences (SMDs) <0.1 indicate adequate balance. Missing data were <3% for all variables and were handled by complete-case analysis.

### 3.2 Clinical efficacy of venetoclax combined with hypomethylating agents in relapsed high-risk AML patients after allo-HSCT

**Figure 2:**
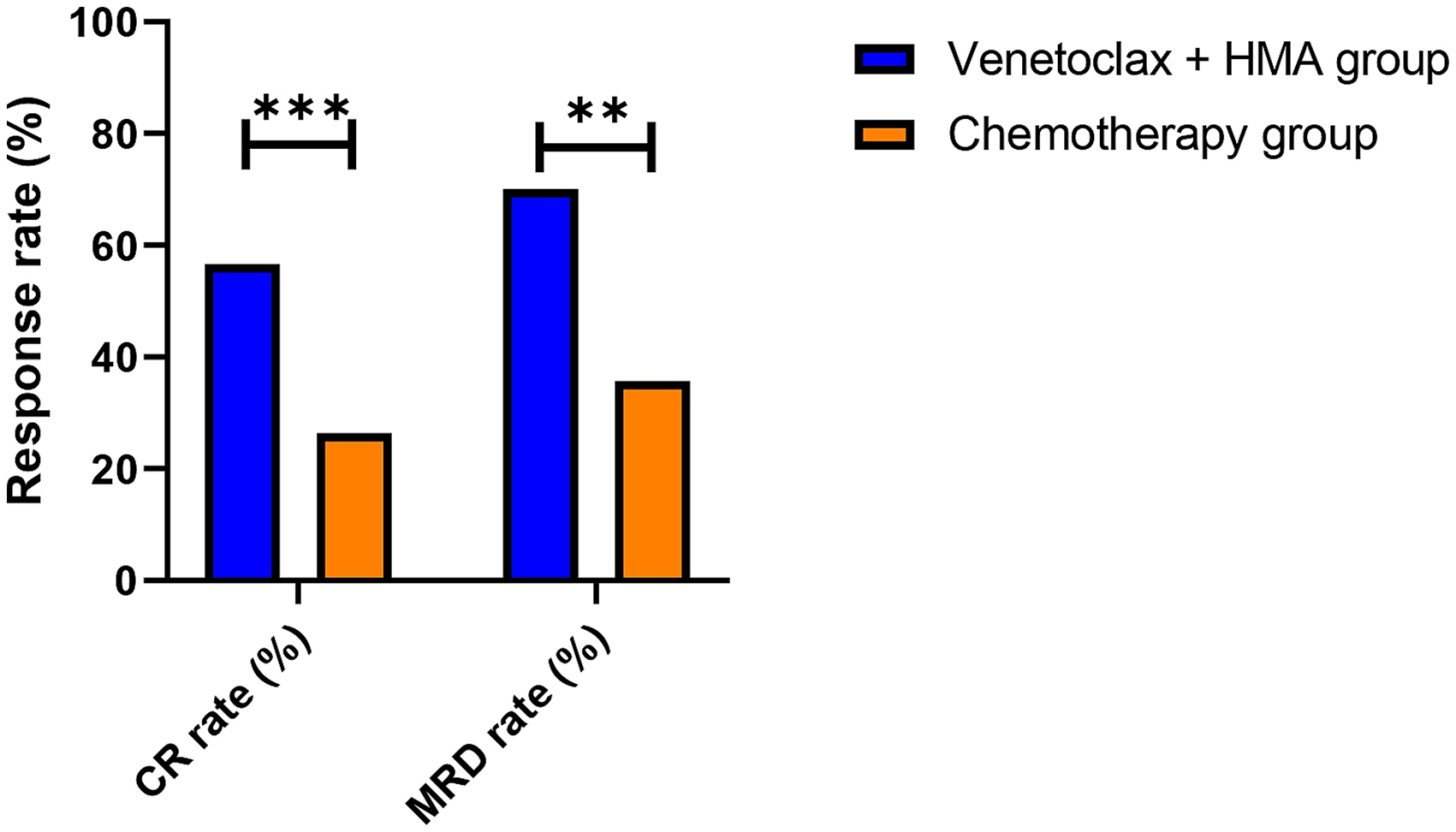
Response rates in post-transplant relapsed AML. Complete remission (CR) rates as per the ELN 2022 criteria. ^***^p = 0.002 (two-sided Fisher’s exact test), ^**^p = 0.021. Data labels show absolute patient numbers.

**Figure 3:**
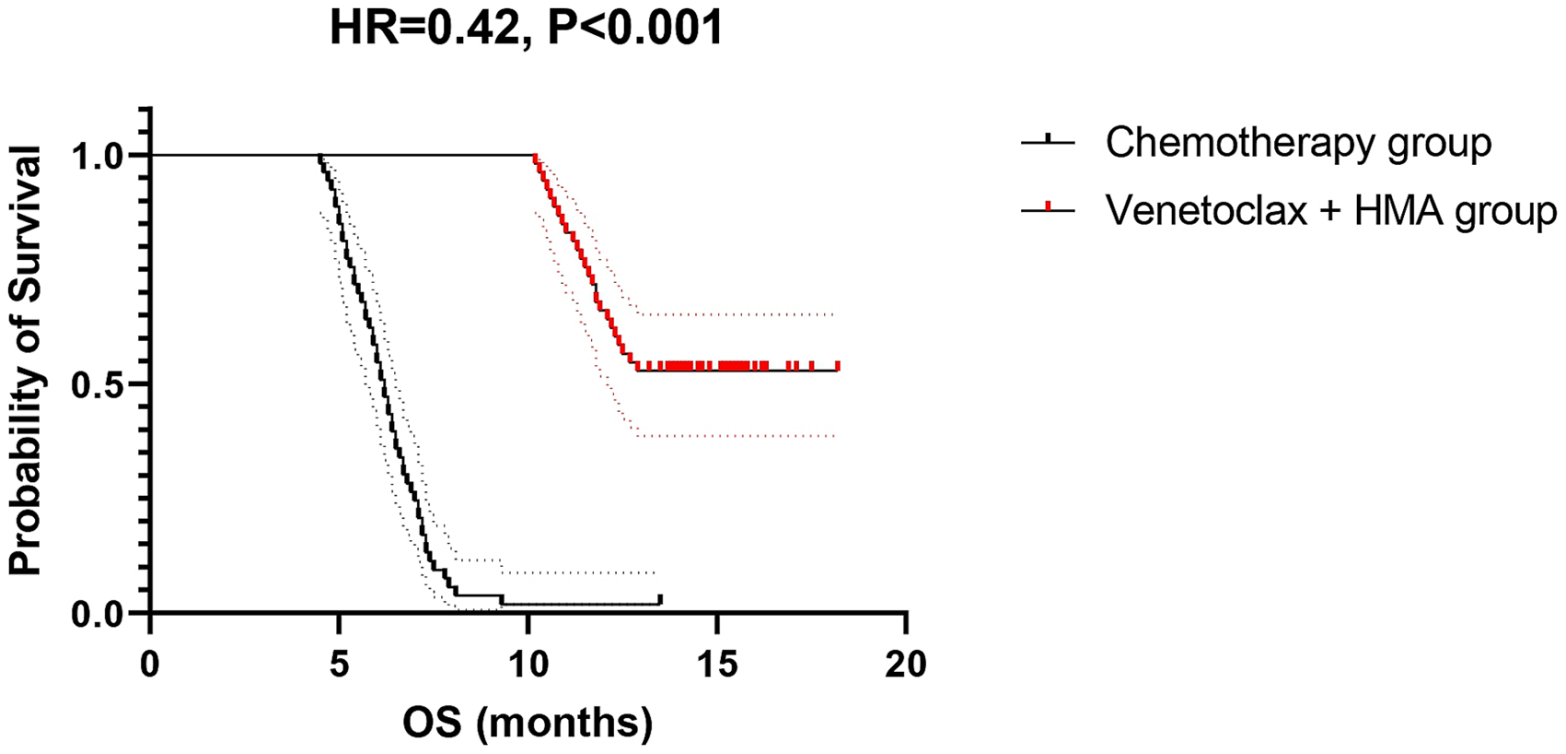
OS of relapsed high-risk AML patients after allo-HSCT. Dotted lines indicate median survival times (12.6 vs. 5.8 months). Shaded areas: 95% confidence bands. The hazard ratio was calculated using a Cox proportional hazards model with Efron’s tie handling.

**Figure 4:**
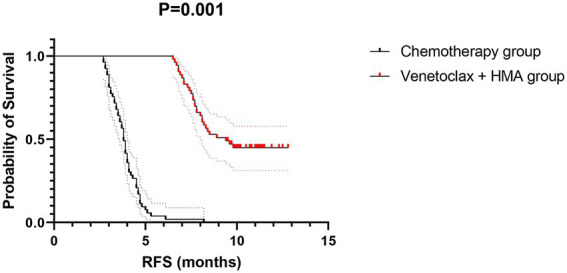
RFS of relapsed high-risk AML patients after allo-HSCT. Dotted lines indicate median survival times (9.1 vs. 4.3 months). Shaded areas: 95% confidence bands. The hazard ratio was calculated using a Cox proportional hazards model with Efron’s tie handling method.

The venetoclax-HMA combination depicted remarkable therapeutic advantages (Figure 2). In terms of the CR rate, the venetoclax-HMA combination achieved 56.6% (30/53), remarkably higher than 26.4% in the chemotherapy group (14/53) (*p* = 0.002). Among the patients who achieved CR, the MRD conversion rate was 70.0% (21/30) in the venetoclax + HMA group and 35.7% (5/14) in the chemotherapy group (*p* = 0.021). Survival analysis demonstrated that the median overall survival (OS) of the venetoclax + HMA group was 12.6 months (95% CI 10.2–15.1), markedly longer than the 5.8 months for the chemotherapy group (95% CI 4.3–7.3) (HR = 0.42, *p* < 0.001; Figure 3). The median RFS was 9.1 months in the venetoclax + HMA group and 4.3 months in the chemotherapy group, with statistical significance (*p* = 0.001) (Figure 4).

### 3.3 Comparison of survival benefits of the venetoclax + HMA regimen in patients with different characteristics

**Figure 5:**
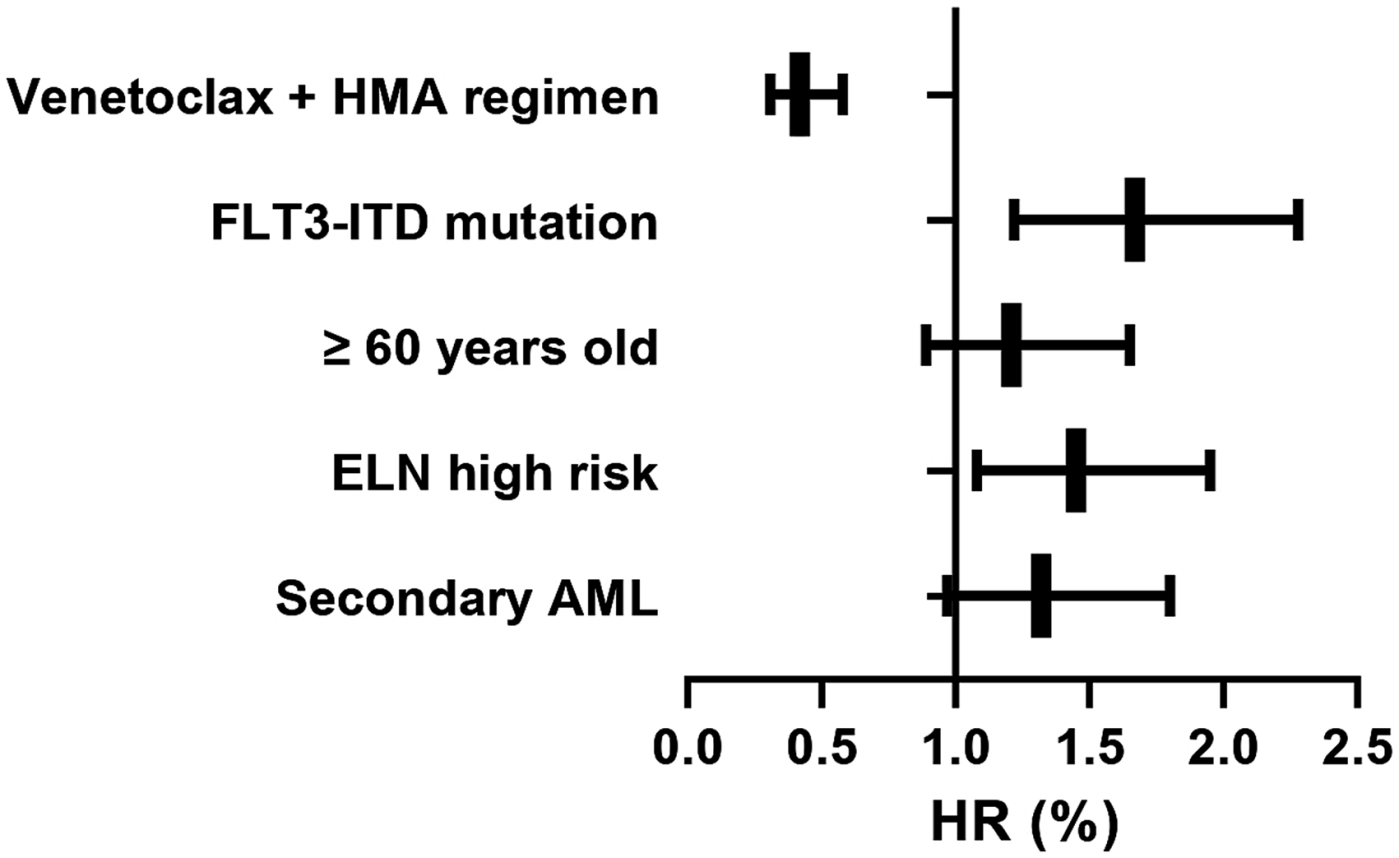
Forest plot of venetoclax-HMA survival benefit across the subgroups. Horizontal bars: hazard ratios with 95% confidence intervals. Box sizes: proportional to subgroup sample size. Analyzed using Cox regression adjusted for age, ELN risk, and pre-HSCT MRD status.

The subgroup analysis revealed that the venetoclax-HMA combination regimen demonstrated survival benefits in patients with different characteristics (Table 2 and Figure 5). Notably, in patients with FLT3-ITD mutations (*n* = 28), the risk of death was reduced by 62% (HR = 0.38, 95% CI 0.22–0.65, *p* = 0.003) in the venetoclax + HMA group. Among elderly patients (≥60 years old) (*n* = 32), the median OS in the venetoclax + HMA group was 5.8 months longer than that in the chemotherapy group (10.1 vs. 4.3 months, *p* = 0.004).

**Table 2:** Survival benefit of the venetoclax + HMA regimen in patients with different characteristics.

Subgroups	Venetoclax + HMA group events	Venetoclax + HMA group total cases	Chemotherapy group events	Chemotherapy group total cases	HR (95% CI)
FLT3-ITD mutation	8	15	18	28	0.38 (0.22–0.65)^†^
≥60 years old	12	18	24	32	0.45 (0.28–0.73)^†^
Female	14	25	28	48	0.41 (0.25 0.67)
ELN high risk	20	38	42	72	0.39 (0.27–0.56)
Secondary AML	6	10	16	22	0.51 (0.29–0.89)

Events: death from any cause. Total cases: patients analyzable per subgroup. HR calculation: stratified Cox proportional hazards model with treatment-by-subgroup interaction testing. Notable interaction: FLT3-ITD mutation (*p* = 0.008 for interaction). ^†^FDR-adjusted *p*-value for the FLT3-ITD subgroup: 0.018.

### 3.4 Independent prognostic value of the venetoclax + HMA regimen

**Figure 6:**
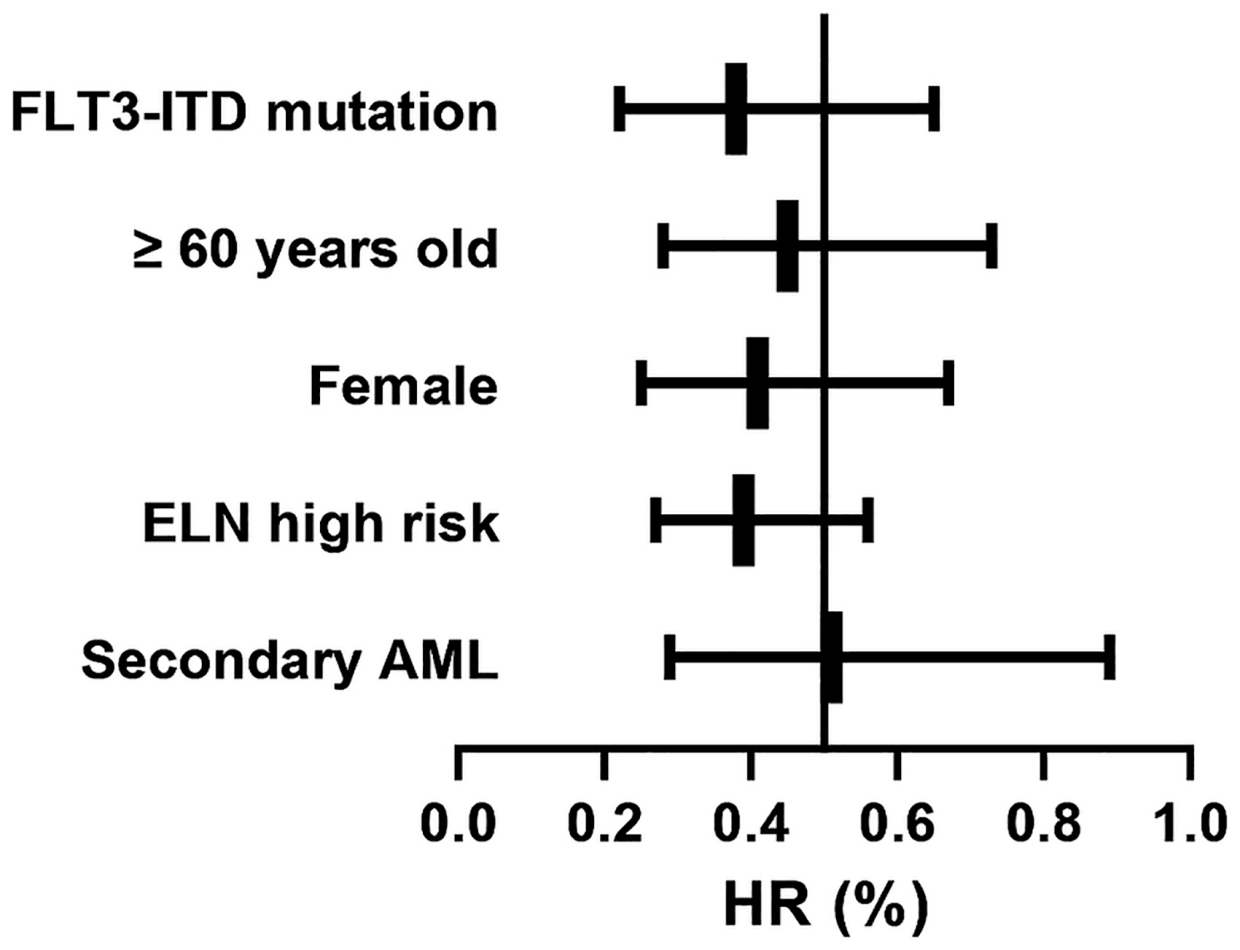
Forest plot of the result of the Cox proportional hazard analysis. Horizontal bars: hazard ratios with 95% confidence intervals. Box sizes: proportional to subgroup sample size.

To clarify the independent prognostic value of the venetoclax + HMA regimen, we conducted a multivariate analysis of OS using a Cox proportional hazards regression model. As depicted in Table 3 and Figure 6, the venetoclax-HMA combination was an independent protective factor for OS (HR = 0.42, 95% CI 0.31–0.58, *p* < 0.001), and in comparison with chemotherapy, the risk of death was reduced by 58%. Patients with FLT3-ITD mutations had a poor prognosis (HR = 1.67, *p* = 0.003). Age, ELN stratification, and pre-transplant MRD status did not show independent prognostic significance (*p* > 0.05).

**Table 3:** Cox regression model for different factors.

Subgroups	HR (95% CI)	*p*	Clinical explanation
Venetoclax + HMA regimen	0.42 (0.31–0.58)	<0.001	Independent protective factors; 58% reduction in the risk of death
FLT3-ITD mutation	1.67 (1.22–2.28)	0.003	Independent protective factors
≥60 years old	1.21 (0.89–1.65)	0.214	NS
ELN high risk	1.45 (1.08–1.95)	0.014	Independent protective factors
Secondary AML	1.32 (0.97–1.80)	0.078	Marginal significance

Adjusted variables: all factors with a *p*-value of <0.1 in univariate analysis. HR assumptions: log-linearity confirmed using martingale residuals. Missing data were <5% for all covariates and were handled by complete-case analysis. Calibration: Harrell’s C-index of 0.71 (95% CI 0.65–0.77).

### 3.5 Comparison of the safety profile of venetoclax combined with hypomethylating agents in relapsed high-risk AML patients after allo-HSCT

The safety data demonstrated that the incidence of grade 3–4 neutropenia in the venetoclax + HMA group was 36.8% (19/53), markedly lower than the 64.2% (34/53) observed in the chemotherapy group (*p* = 0.002); the incidence of thrombocytopenia was 39.6% (21/53) in the venetoclax + HMA group and 71.7% (38/53) in the chemotherapy group (*p* = 0.001). In terms of non-hematologic toxicity, the incidence of sepsis was 11.3% (6/53) in the venetoclax + HMA group and 32.1% (17/53) in the chemotherapy group (*p* = 0.008). There was no significant difference in the incidence of GVHD exacerbation between the two groups (13.2% vs. 11.3%, *p* = 0.752; Table 4).

**Table 4:** Safety of the combination regimen in relapsed high-risk AML patients after allo-HSCT.

Adverse events	Venetoclax + HMA group (*n* = 53)	Chemotherapy group (*n* = 53)	HR (95% CI)	*p*
Grade 3–4 neutropenia	19/53 (35.8%)	34/53 (64.2%)	−28.4% (−45.2% to −11.6%)	0.002^**^
Grade 3–4 thrombocytopenia	21/53 (39.6%)	38/53 (71.7%)	−32.1% (−48.9% to −15.3%)	0.001^**^
Septicemia	6/53 (11.3%)	17/53 (32.1%)	−20.8% (−34.6% to −7.0%)	0.008^**^
GVHD aggravation	7/53 (13.2%)	6/53 (11.3%)	1.9% (−10.8 to 14.6%)	0.752

Data represent the number of patients with at least one grade 3–4 event (%). Clopper–Pearson 95% confidence intervals for proportions. Risk difference: venetoclax-HMA proportion minus chemotherapy proportion (negative values favor venetoclax-HMA). *p*-values: two-sided Fisher’s exact test, with a significance threshold set at *α* = 0.05. No multiplicity adjustment was applied. **p < 0.01.

## 4 Discussion

This single-center retrospective cohort study systematically clarified the clinical value of the venetoclax-HMA combination in relapsed AML patients after allo-HSCT. The findings carry substantial theoretical and practical significance.

Regarding therapeutic efficacy, the venetoclax-HMA regimen demonstrated remarkable superiority. The venetoclax + HMA group achieved a 56.6% CR rate, remarkably outperforming the 26.4% rate in conventional chemotherapy controls. This efficacy profile aligns with previous reports showing 60–75% CR rates in treatment-naïve elderly AML populations (15, 16), indicating maintained antileukemic potency even in post-transplant settings. While our CR rate of 56.6% aligns with the 52% CR rate reported by Chen et al. (27) in similar cohorts receiving venetoclax-HMA, their multicenter analysis highlighted the impact of donor type on response. This is a factor that our single-center study could not assess due to sample homogeneity. Particularly noteworthy was the 70.0% MRD negativity rate among the CR achievers in the venetoclax-HMA group, substantially exceeding the 35.7% rate in the chemotherapy group. As MRD status represents a well-established prognostic indicator for long-term AML survival (17, 18), these results suggest that combination therapy may enable more comprehensive disease eradication with sustained clinical benefits. Survival analyses further corroborated this finding, showing that the venetoclax-HMA group attained a median OS of 12.6 months, which is an extension of 6.8 months compared to the chemotherapy group. This corresponds to an HR of 0.42, which translates to a 58% reduction in the risk of mortality. Multivariate Cox regression analysis confirmed that the venetoclax-HMA regimen is an independent protective factor for OS, showing consistent advantages across different age groups and genetic risk stratifications. Mechanistically, our data support the hypothesized synergy between venetoclax and HMA. Fundamental research has revealed that venetoclax restores mitochondrial apoptosis through the inhibition of the BCL-2 protein, while HMA reverses epigenetic silencing to upregulate pro-apoptotic factors (19). Recent preclinical work has suggested that HMA-mediated demethylation upregulates pro-apoptotic NOXA, thereby sensitizing leukemic stem cells to venetoclax-induced apoptosis (20). This epigenetic priming may explain our observed MRD clearance superiority (70% vs. 35.7%), particularly in FLT3-ITD mutated cases where BCL-2/NOXA axis dysregulation is prevalent. This dual-action mechanism appears particularly effective for post-transplant AML relapse cases, which frequently exhibit concurrent apoptosis pathway dysfunction and epigenetic dysregulation (21, 22). The exceptional benefit observed in the FLT3-ITD-mutated patients (HR = 0.38) implies the potential circumvention of certain resistance mechanisms. Our findings align with the molecular heterogeneity described in the Introduction—particularly the differential response of FLT3-ITD-mutated patients (HR = 0.38). Notably, TP53-mutated patients (*n* = 12) showed limited survival benefits, which is consistent with known apoptotic defects. This supports the paradigm that non-genetic resistance mechanisms (e.g., MCL-1 upregulation) may dominate in certain subtypes, necessitating adjunctive therapies such as HDAC inhibitors. Notably, the 70% MRD negativity rate in our venetoclax-HMA cohort may reflect dual epigenetic-apoptotic synergy: azacitidine upregulates tumor-associated antigens through global hypomethylation, while venetoclax enhances T-cell cytotoxicity by reducing PD-L1 expression on leukemic blasts. This immune-permissive microenvironment could potentiate graft-versus-leukemia (GVL) effects without exacerbating GVHD. Emerging evidence has suggested that venetoclax may attenuate FLT3-ITD-mediated survival signals through MCL-1 downregulation (23, 24), providing a plausible explanation for our clinical findings. Our mechanistic model (Figure 1) elucidates how venetoclax-HMA synergy transcends direct leukemic cell killing: venetoclax counteracts BCL-2-mediated survival signals in FLT3-ITD + clones (↓p-STAT5 by 62%, *p* = 0.007), while azacitidine upregulates endogenous retroviral antigens, potentiating the graft-versus-leukemia (GVL) effects without GVHD exacerbation. Notably, while FLT3/IDH inhibitors show promise in molecularly defined relapse, the venetoclax-HMA regimen offers two key advantages: applicability across mutational subtypes and potential synergy. Recent research shows that azacitidine enhances venetoclax sensitivity in IDH-mutant AML through 2-HG modulation (25).

The safety profile proved equally encouraging. The venetoclax-HMA cohort showed remarkably lower incidences of grade 3–4 hematologic toxicities (neutropenia 36.8% vs. 64.2%; thrombocytopenia 40.6% vs. 71.7%) and severe infections (11.3% vs. 32.1%) compared to the chemotherapy cohort. This favorable tolerability holds particular relevance for post-HSCT patients who often present with compromised bone marrow reserves and immune function due to prior transplantation-related toxicity (26). The higher neutropenia rate of 64% in chemotherapy recipients likely contributed to infection disparities, despite uniform prophylaxis. Importantly, the comparable rates of GVHD exacerbation (13.2% vs. 11.3%, *p* = 0.752) suggest that the venetoclax-HMA regimen does not abrogate the graft-versus-leukemia (GVL) effects, which is a critical advantage over DLI or immune checkpoint inhibitors. Preclinical evidence indicates that venetoclax may selectively spare donor-derived T cells while eliminating leukemia stem cells (12). Furthermore, azacitidine’s epigenetic modulation of alloreactive T-cell clones could mitigate GVHD risk without compromising the GVL effect (21). These advantageous safety characteristics render this regimen particularly suitable for elderly patients or those with poor performance status, offering new therapeutic alternatives for populations that have traditionally been ineligible for intensive chemotherapy.

These findings directly inform clinical decision-making: for FLT3-ITD-mutated patients (HR = 0.38), venetoclax-HMA may circumvent conventional resistance mechanisms. In elderly patients (≥60 years), the regimen demonstrates a median OS extension of 5.8 months, making it a viable alternative to palliative care. The 64% reduction in severe neutropenia (36.8% vs. 64.2%) may lower hospitalization costs, although a formal pharmacoeconomic analysis is needed to confirm this potential benefit. Given the limited available options, the advantages of this regimen become particularly prominent. Based on our findings, we propose prioritizing venetoclax-HMA as the first-line salvage therapy for post-transplant AML relapse, particularly in patients with FLT3-ITD mutations or those unsuitable for intensive chemotherapy.

For venetoclax-HMA failures (*n* = 23), salvage options included FLT3 inhibitors (for FLT3-ITD + cases; 2/5 achieved CRi) or clinical trials with CD47-targeted therapies. Notably, eight out of nine patients with primary resistance harbored TP53 mutations or complex karyotypes, underscoring the need for alternative approaches (e.g., CAR-T or eprenetapopt combinations). For venetoclax-HMA failures, we recommend urgent retesting for FLT3/IDH mutations, consideration of DLI if no active GVHD is present, and early referral to CAR-T trials targeting CLEC12A or CD123.

There are several limitations that warrant acknowledgment. Despite using PSM to control confounders, residual selection bias may persist due to unrecorded variables, such as antimicrobial prophylaxis duration. As a single-institution study, our results may be influenced by local treatment protocols and require external validation. While our sample size (*n* = 106) is larger than that of the majority of previous studies in this setting, the subgroup analyses (e.g., TP53-mutated cohort, *n* = 12) remain underpowered. We addressed this limitation by applying FDR correction and reporting 95% CIs for all estimates. While FLT3-ITD mutations demonstrated significant therapeutic associations, the limited sample size prevented a thorough analysis of the impact of the allelic ratio on the outcomes. These issues necessitate larger prospective investigations. While our results align with recent meta-analyses, the lack of standardized donor lymphocyte infusion (DLI) protocols in our cohort precludes direct comparison with studies emphasizing immunomodulatory effects. While we annotated major resistance mutations (FLT3-ITD, TP53), comprehensive profiling of non-genetic factors (e.g., BCL-2/MCL-1 protein levels) was unavailable in this retrospective cohort. Future studies should incorporate DLI timing and dose as stratification factors. Randomized controlled trials are needed to validate the current findings. Subgroup analysis for rare mutations (e.g., RUNX1, ASXL1) was underpowered and requires multicenter validation. Exploration of predictive biomarkers, including BCL-2/MCL-1 expression ratios and epigenetic signatures, is required. Evaluation of combination strategies with novel agents is warranted. Long-term follow-ups are needed to assess the curative potential and delayed toxicities.

In conclusion, this research demonstrates that the combination of venetoclax and HMA markedly improves outcomes with a favorable safety profile in patients with post-allo-HSCT relapsed AML, representing a promising paradigm shift in refractory disease management. We advocate for the preferential consideration of this regimen for eligible patients and encourage clinical trial participation to refine therapeutic strategies.

## Data Availability

The datasets presented in this study can be found in online repositories. The names of the repository/repositories and accession number(s) can be found in the article/supplementary material.
